# ‘Coz football is what we all have’: masculinities, practice, performance and effervescence in a gender‐sensitised weight‐loss and healthy living programme for men

**DOI:** 10.1111/1467-9566.12402

**Published:** 2016-02-11

**Authors:** Christopher Bunn, Sally Wyke, Cindy M. Gray, Alice Maclean, Kate Hunt

**Affiliations:** ^1^ Institute of Health and WellbeingUniversity of GlasgowScotlandUK; ^2^ MRC/CSO Social and Public Health Sciences UnitUniversity of GlasgowScotlandUK

**Keywords:** masculinity/masculinities, obesity, durkheim, football, health practices, men's health

## Abstract

In this paper we use a social practice approach to explore men's experience of Football Fans in Training (FFIT), a group‐based weight management programme for men that harnesses men's symbolic attachment to professional football clubs to engage them in lifestyle change. FFIT is delivered by community coaches in clubs’ stadia and is gender‐sensitised in relation to context, content and style of delivery. Using a ‘toolkit’ of concepts from the work of Bourdieu, Goffman and Durkheim we analysed data from 13 focus group discussions with participants, and fieldwork notes from programme observations to investigate the appeal and success of FFIT, and how it worked to support change. Our analysis builds on our work on the importance of shared symbolic commitment to the football club and being with ‘men like me’ to understand how the interaction context facilitated ‘effervescent’ experiences. These experiences encouraged men to make changes to their diet and physical activity, talk about them, practice performing them and implement them in their lives. Thus a social practice approach illuminated the social processes through which lifestyle change was achieved, and we argue that it can deepen and enrich both intervention design and evaluation.

## Introduction

Scrutiny of various ‘health behaviours’ has intensified in recent years as public health researchers and practitioners have sought to intervene to change them. Despite recent advances in behavioural theory and in specifying behaviour change techniques in intervention development (Michie *et al*. 2013), sociological thought is seldom integrated. Yet, everyday behaviours such as the consumption of food and drink, engagement in physical activity and time spent sedentary are all practical and cultural phenomena that simultaneously reflect our positions in social space (Bourdieu 1984), and influence our body shape, morphology and function, and so our risk of morbidity and mortality. Nettleton and Green (2014) have argued that discourses of behaviour change often over‐emphasise the rationalised accounts of behaviour provided by research participants, at the expense of exploring the tacit knowledge that constitutes much social practice, such as what, when and how we ‘choose’ to eat. The Bourdieuian sociology of practice (Bourdieu 1977) they draw on emphasises the multi‐dimensionality of human action: behaviours such as the consumption of alcohol are not simply the product of decontextualised ‘choices’, but they become embodied and enacted through performances that reflect the cultural roles and narratives that we learn as we grow up and move between social spaces (Lyons et al. 2014). There is thus an ongoing challenge for social and behavioural scientists to more comprehensively integrate multidisciplinary perspectives on the practices and theories of health behaviour change, and particularly for sociologists of health and illness (Cohn 2014), who seek to understand the social conditions which facilitate, inhibit or prevent particular health practices. The challenge, as we see it, is for sociologists to contribute understandings of how social practices are made and re‐made in interactions, and how these practices then become established as ‘tacit knowledge’, namely, as behaviours that are largely accepted, and seldom confronted or challenged.

The importance of understanding these practices is relevant for public health challenges such as rising obesity rates (Wang *et al*. 2011). The development of effective and acceptable weight management interventions to support people who wish to lose weight needs to be cognisant of relevant social practices if behaviour change is to be sustainable. These social practices are defined as much by constraints imposed by the routines and orthodoxies of social and cultural environments as they are by the cognitive processes that influence behaviours. With this in mind, the study of, and intervention in, the obesity ‘epidemic’ is amenable to a social practice‐based approach to health‐related action that focuses on how the actor negotiates identity from a specific point in social space.

As many of the behaviours which contribute to weight gain and weight management are related to gender, the obesity ‘epidemic’ poses some different challenges for men's and women's health; in this paper we focus on men. The notion of multiple masculinities has informed much recent work on men's health, including our own. Although contested, recognition of ‘global’, and particularly ‘regional’ (‘constructed as the level of the culture or nation‐state’) and ‘local’ (‘constructed in the arenas of face‐to‐face interactions of families, organizations, and immediate communities’) forms of hegemonic masculinity that provide ‘a cultural framework that may be materialized in daily practices and interactions’ (Connell and Messerschmidt 2005: 849‐50) are useful in framing and reframing understandings of men's health behaviours in everyday life. In Scotland, where the study we report here was conducted, fewer than half of men (45%) meet national physical activity recommendations and 69% are overweight or obese (Bradshaw *et al*. 2012). Men are under‐represented in commercial and health services‐based weight‐management services (Robertson *et al*. 2014), probably reflecting, at least in part, dominant constructions of masculinities that cast ‘dieting’ as feminine (Gough 2007). In addition, it has been suggested that men prefer individual to group approaches to weight management (Lubans 2014, Morgan *et al*. 2013) which might further limit the appeal of most group‐based programmes.

One approach to engaging men in health‐related activities has been to base them within football (Pringle *et al*. 2011) or other professional sports settings (Witty and White 2011). A guiding principle for one such intervention that we developed (Gray *et al*. 2013) and evaluated (Hunt *et al*. 2014b) (Football Fans in Training (FFIT)), was that, by offering programmes within the traditionally male‐dominated environment of the professional football club, the threats that ‘weight‐management’ programmes may pose to men's ‘masculine capital’ (de Visser and McDonnell 2013), might be offset by enhanced physical and symbolic proximity to the club and fellow male supporters (Hunt et al. 2014a). Hence, we consciously aimed to exploit multiple hierarchies of masculinity. Sports‐settings and sporting ability (and ‘skilled bodily activity’ (Connell and Messerschmidt 2005: 851)) are intimately tied to constructions of masculinity in many cultures (as discussed below), and commercial sports are a ‘focus of media representations of masculinity’ (Connell and Messerschmidt 2005: 833). Indeed, recent studies show that football clubs are perceived by many men as attractive settings for health interventions (Gray *et al*. 2013, Hunt *et al*. 2014a, Pringle *et al*. 2013, Zwolinsky *et al*. 2013), and that social interactions within such programmes can support the pursuit of healthier lifestyles (Robertson *et al*. 2013).

The use of professional football clubs as sites for the delivery of men's health interventions has, however, also been problematised. In an evaluation of a football‐based mental health programme, Spandler and colleagues noted how men who attended simultaneously spoke in ways which challenged and reproduced hegemonic formulations of masculinity (Spandler *et al*. 2014). In earlier work, Spandler and McKeown (2012: 401) characterised football‐based interventions as ‘paradoxical social spaces’ in which traditional and ‘oppressive’ gender relations can be both reproduced and challenged; ‘it is possible for them to act as a forum that consciously challenges masculine hegemony’. In the same paper they also contend that football‐related health initiatives can:

Consciously develop countercultural space, which do not merely reflect and reproduce dominant sports cultures, but actually build in reflection and contestation. In this way, it may be possible to turn the contradictions, complexities, and ambiguities within football (and gender) into important topics for discussion and intervention (Spandler and McKeown 2012: 401).

Elsewhere we have discussed how theories of masculinities have informed our understanding of health behaviours as performances of gender, and hence the development and evaluation of FFIT (Gray *et al*. 2013, Hunt *et al*. 2013, 2014b). In this paper, we draw on sociological theories of practice (Bourdieu), performance (Goffman) and collective effervescence (Durkheim) to investigate further the appeal of FFIT and how it worked to support changed lifestyles. To do this we use qualitative data gathered as part of the process evaluation embedded within a randomised controlled trial (RCT) of the effectiveness of the programme. Below we present a brief overview of FFIT and findings from the RCT (and related work) before discussing these social theories. After describing our analytic approach and the data through which we develop our arguments, we consider how a focus on interaction and practice has the potential to play a role in public health initiatives and the human actions on which they are based.

## The football fans in training programme

The FFIT programme was based on best evidence of what works for weight loss (SIGN 2010); the development of the programme is fully described in Gray *et al*. (2013). FFIT is a group‐based, 12‐week, weight management, physical activity and healthy living programme delivered by club community coaches in professional football clubs. The delivery of FFIT in Scotland is co‐ordinated by the Scottish Professional Football League (SPFL) Trust, with whom we collaborated throughout programme development and evaluation. Each session includes a ‘classroom’ component, followed by group physical activities led by club community coaches, with each man encouraged to work at a level of exertion appropriate to his own health and fitness. The programme is gender‐sensitised in: context (football club setting, men‐only groups); content (e.g. information on the science of weight loss presented simply – sometimes described by the men as ‘science but not rocket science’, discussion of alcohol in relation to weight management, and club ‘branding’ of FFIT materials); and delivery style (participative, peer‐supported, learning which encourages interaction and ‘banter’ to facilitate discussion of sensitive subjects) (Wyke *et al*. 2015).

The FFIT programme has successfully attracted overweight and obese men aged 35–65 years, a group at enhanced risk of future disease (Hunt *et al*. 2014a). To evaluate the programme we conducted a RCT (Hunt *et al*. 2014b, Wyke *et al*. 2015) which demonstrated that mean difference in weight loss 12 months after baseline between men who had done the programme and a waitlist comparison group who had not yet done FFIT, adjusted for baseline weight and club attended, was 4.94 kg (95% confidence intervals 3.95, 5.94) in favour of the intervention group (Hunt *et al*. 2014b). Findings from the process evaluation have been reported elsewhere (Wyke *et al*. 2015).

## Practice, performance and effervescence: a sociological toolkit

Bourdieu's sociology of practice emphasises the importance of social environment and interaction in the accumulation of experiences that shape actions. Bourdieu's simple but powerful statement that ‘the social world is accumulated history’ (Bourdieu 1986: 46) undergirds the concepts of habitus, field, capital and strategy, which are often considered to be the cornerstones of his conceptual ‘toolkit’. Many excellent summaries of these concepts have been published (e.g. Williams 1995). In brief, and in relation to the arguments we make in this paper, the *habitus* is the site in which social and cultural experiences collect, providing actors with the resources to judge and generate actions. In Bourdieu's words the habitus is ‘embodied history, internalized as a second nature and so forgotten as history – the active presence of the whole past of which it is the product’ (Bourdieu 1990a: 56). For this reason, the habitus is largely pre‐conscious, structured by an unthinkably large amount of experience. Past experiences form the basis on which actors are able to apprehend the ‘rules’ or ‘orthodoxies’ of the social contexts in which they find themselves interacting. Bourdieu describes this as *‘*doxic experience’, which he characterises as a ‘feel for the game’ and, again, as a tacit and pre‐conscious aspect of human action (Bourdieu 1990b: 61). Bourdieu also developed the concept of ‘hexis’ to refer to the embodied aspects of habitus and doxa: ‘a permanent disposition, a durable manner of standing, speaking and thereby feeling and thinking’ (Bourdieu 1977: 93–4). Hexis describes the historically, and biographically, mediated and stylised ways in which bodies are used in interactions.

The relevance of these concepts to the data we present can be illuminated by Swain's research on the socialisation experiences of boys. Swain's investigation of the role played by football in the construction of masculinities has suggested that, through participating in playground matches, boys:

Were practising to become men. By the time they have reached the age of 10 or 11, many of the boys will have spent thousands of hours, almost in rehearsal, of trying to look like, and emulate, their professional heroes (Swain 2000: 101).

Swain suggests that, in these playground encounters, boys live out ‘the ideals of fitness, strength, competition, power and domination’ and so begin the process of inculcating practices associated with dominant masculinities (Swain 2000: 107). This interpretation fits a Bourdieuian account of the development of habitus, doxa and hexis. Through playground interactions, and the cultural symbols and doxa of professional football that they are constructed in relation to, the boys Swain observed learnt to construct a tough bodily hexis that, for example, reinforced that they should not cry when they were punched. Within such encounters, as well as through the obsessive following of football stars and their teams, Swain's footballing boys shaped their doxa, hexis and, ultimately, their habitus by internalising socially‐mandated bodily behaviours: ‘the child imitates not ‘models’ but other people's actions’ (Bourdieu 1977: 87). Even boys that did not play football, Swain notes, were influenced by this performance of masculinity, as its dominance meant that it became the symbolic form through which much masculine action was appraised.

The habitus, doxa and hexis learnt through childhood experiences (such as playground football or, indeed, exclusion from playground football, with their emphasis on masculine power and assertion), influence the practice of ‘manhood’ in adult life. A complementary focus on social performance, based on Goffman's dramaturgical approach to social interaction and identity performances, can illuminate how masculinities, and the practices through which they are realised, can shift. For Goffman, interaction is characterised by the need to establish a definition of the situation at hand; to construct a ‘working consensus’ between actors within an encounter (Goffman 1959). During an interaction, and whilst seeking to establish consensus, actors attempt to manage the impressions they give to others by performing socially‐acknowledged ‘roles’ and using a ‘repertoire’ of ‘fronts’ that fit these. In the literature on men's health, it is often argued that performances of hegemonic masculinities encourage health‐damaging practices. For example, Courtenay claimed that dominant male roles encourage men to embrace risky habits (e.g. excessive drinking or smoking), and poor self‐care (e.g. lack of sleep, poor diet, denying stress); in his words, ‘when a man brags, “I haven't been to a doctor in years”, he is simultaneously describing a health practice and situating himself in a masculine arena’ (Courtenay 2000: 1389).

Others, for example de Visser and colleagues (2009), have drawn on Bourdieu's notion of symbolic capital to argue that men can ‘offset’ negative appraisals of their masculinity (brought about, for example, by not drinking alcohol) by emphasising their masculine capital in other domains (for example, being known to have great sporting prowess). Similarly, O'Brien and colleagues (2005) argued that, whilst men recognised a dominant cultural discourse that dictates that men ‘should’ appear reluctant to consult with a doctor, this could be set aside to preserve other, more valued, masculine attributes; hence men described readily seeking help in order to stay healthy enough to retain a stereotypically masculine occupation (e.g. firefighter) or to maintain sexual function. To conceptualise such performances of resistance, in which men seek to negotiate the legitimacy of hegemonically disparaged activities, we could extend Goffman's model by including de Certeau's (1984) notion of ‘tactics’: the practices through which dominance is resisted and autonomy is partially re‐claimed). As the studies mentioned above demonstrate, hegemonic masculinity can be disrupted, when a ‘repertoire’ of ‘tactical performance’ is deployed to gain respect and recognition for prioritising alternative forms of masculine capital.

Theories of practice and performance, as discussed above, describe how social orthodoxies are reproduced in interactions through the deployment of the embodied resources of habitus, doxa and hexis. In terms of masculinities, we have argued that viewing performance through the lens of what we might term ‘tactical repertoire’ can demonstrate how individual men can resist or redefine ‘hegemonic masculinities’ that encourage ‘toxic’ practices (Connell and Messerschmidt 2005; Emslie *et al*. 2006). However, practice and performance‐based theories do not straightforwardly allow us to examine how such masculinities are re‐produced on a larger scale, for example, at group or societal levels.

Sociological accounts of sport, and football in particular, shed light on how hegemonic masculinities can be reproduced at these collective levels. For example, in his ethnographic account of supporters of Millwall Football Club (FC), Robson shows how ‘the club's cultural community is bound together by specific understandings of class, masculinity and local history’ (Robson 2000: 7–8). These bonds, he argues, make Millwall FC more than a sporting club, transforming it into a locus of the ‘collective imaginary’ through which supporters perform and (re‐)produce their identities. The force of these identity processes, Robson claims, is brought to bear by the ritualised nature of the practices within the Millwall community: attending matches, singing songs and exchanging experiences with fellow members of the ‘Millwall collective’. These have the effect of establishing a ‘deeply personal … fusion of symbol and self’ which leaves the supporter ‘charged up, confirmed and strengthened’ (Robson 2000: 185).

Arguments, such as Robson's, that emphasise the importance of ritual practices amongst football supporters draw heavily on the Durkheimian school of cultural analysis. In his seminal analysis of religious culture and ritual, Durkheim claimed that acts of religious ritual and worship are moments of socio‐genesis (Durkheim 1926, Lash 2010). During such rituals, participants allow a symbolic fusion of self and collective symbols to take hold of them, as Robson notes of Millwall fans. For Durkheim, and implied in Robson's account, these moments of ‘fusion’ take place through ‘collective effervescence’: of group narration, singing, dancing and moving (Durkheim 1926: 284). Through repetitive practice of such collective rituals, an individual, in this case the football fan, assimilates the symbolic power of the football club into his/her own internalised schemes of perception, their ‘habitus’. For Durkheim (1926: 157), such processes of assimilation mean that the participant subsequently ‘approaches the world with confidence and the feeling of energy’.

Below we illustrate how the sociological theories described above can inform our understanding of the appeal and success of the FFIT programme and how it worked to support lifestyle changes, through an analysis of two sets of data: observations of delivery sessions of FFIT and focus group data on men's experiences of participating in the programme. Specifically, we use the ideas outlined to explore how participants responded to each other in group settings to negotiate and perform new health practices. We return to these ideas in the discussion and reflect on how they might inform the development of public health interventions.

## Methods

Our two data sources were gathered as part of the process evaluation of FFIT embedded within the RCT of its effectiveness. First, semi‐structured focus group discussions were conducted with 63 men at the 13 participating clubs soon after they had completed the programme. When contacted about the focus groups, 133/295 (45%) of those approached expressed an interest in taking part. All focus groups were videoed and audio‐recorded with participants’ permission, and were transcribed and anonymised. Transcripts were checked for accuracy against the recordings.

Second, observations of 26 of the 156 sessions that were delivered during the FFIT RCT were undertaken. Observations were sampled to ensure that each club and each weekly session was observed twice. The ‘classroom’ component of each observed session was audio‐recorded and the physical activity component was videoed. These digital data were used during the writing‐up of field notes to supplement written notes made on a structured proforma by the researcher observing the session. Observation notes focused on: the extent to which coaches delivered each session's key tasks as specified in the programme delivery guide; group‐based factors (e.g. interactions between participants, and between participants and the coaches); and identification of examples of particularly good practice and/or any problems/issues of programme delivery.

NVivo 10 (QSR International, Brisbane) was used to facilitate systematic approaches to the coding and retrieval of data related to anticipated and emergent themes. First, we used a thematic approach to identify broad themes (Guest *et al*. 2011). Data coded to each theme were subsequently independently coded for sub‐themes by two researchers who then met to discuss their readings of the data and agree a final coding scheme. A recurrent finding that emerged from both focus group and observation data was the significance placed by participants on the quality and nature of the interactions between men during the FFIT sessions. Data relating to these interactions were extracted and analysed by one researcher in an iterative way, drawing on social theory. This approach deployed theory as a set of ‘thinking tools’ which are made ‘visible through the results they yield’ i.e. the narrative argument constructed below (Bourdieu and Wacquant 1992: 160).

Ethical approval for the evaluation (ISRCTN32677491) was received from the University of Glasgow's College of Social Sciences Ethics Committee (CSS/2011/029), which complies with the UK Economic and Social Research Council's Framework for Research Ethics. Participants and coaches provided written informed consent prior to each data collection point, were assured their data would remain anonymous and were aware that they were free to withdraw from the research at any time (Hunt *et al*. 2014a, 2014b)

## Findings

### ‘Three, four, five things in common’

One recurrent finding emerging from analysis of the focus group data was the value placed on being with other men ‘like’ them whilst on the FFIT programme. We have reported the central role that these (assumed) commonalities played in relation to men's initial engagement with FFIT elsewhere (Hunt *et al*. 2014a). However, it is necessary for our argument here to reiterate the ways in which men articulated being with other men whom they saw as being sufficiently ‘like them’; men with similar (enough) bodies and of a similar (enough) age, facing similar weight loss challenges, who were also football fans (and usually supporters of the same club). The following exchange illustrates this:

P1 We're all similar, old, fat gits.

P6 Yeah, exactly.

P1 You know? And that was it.

P5 You could count five points we all have in common, right? One – age. One – weight, yeah? [Club03] supporters, yeah? I've ran oot [out] of ideas on that.

P6 All want to lose weight, all want to get fit.

P4 Uh huh.

P1 Male.

P2 Hair falling out.

P5 Yeah.

P1 No, mine's fell out.

P5 Every single one of us (overtalking) has sort of, ok, I'll say three, four, five things in common, and that's the pulling thing for the whole lot. [Club03_12wkFGD]

Participants’ shared interest in football, and often specifically their own club, was frequently observed to strengthen group interaction and encourage exchanges, both by men themselves and by the researchers observing FFIT sessions. Observation notes of one session record that ‘one man had brought in old photos of [stadium] to show to [the coach]. This created a lot of chat’ [Club08_ObsWk2].

The shared cultural reference point of the club stimulated conversations and contributed to the emergence of group interactions that confirmed the perception for members that they were amongst men ‘like them’. In Bourdieuian terms, we suggest that they recognised aspects of shared habitus; a similar set of accumulated experiences of supporting the same football club, of body, of age, of gender, which men recognised through the kinds of conversations and actions that were generated in the group interactions that took place during FFIT.

Thus the sharing of similar cultural reference points and investments as others in the group was seen as supportive of the pursuit of the shared goal of weight loss. This point was also narrated with reference to the absence of other men (and women) seen as too threatening to such pursuits. For example, in focus group discussions:

P1 I think it was quite good because … you werenae [weren't] gonna have your twenty‐year‐old who could, maybe is slightly overweight, who'll find it easy to lose where, when you're a wee bit older, it's a lot harder. [Club08_ 12wkFGD]

P6 I think it was good because it meant that you were going to come along here and it wasn't going to be like a gym, where they're all Adonises. It was blokes like yourself who'd let themselves go a bit and wanted to do something to get back a bit. And I think that was the clincher, for me, that I wasn't going to feel out of place. [Club13_12wkFGD]

Men's talk in the groups was thus suffused with implicit and explicit references to what they implicitly perceived as hegemonic forms of masculinity, especially as inscribed in the body. The fact that the men felt comfortable in the company of men whom they assumed were not going to out‐perform them (i.e. were not younger, finding it easy to lose weight, or ‘Adonises’) demonstrates the significant hold that the embodied hegemonic masculine values of competition and prowess can have on self‐image and routine practice. By attracting men with ‘three, four, five things in common’, and by creating a space in which the bodily hexis of their peers was not threatening, FFIT was able to defuse some of the fears that men's doxic experience of inter‐male interactions often generates.

In summary, the feeling of being amongst other men with a shared cultural investment, of a broadly similar age and with similarly unsculpted bodies was seen to be important. These commonalities facilitated the fostering of an environment in which the worst excesses of masculine forms of competition, which men likely learnt in the school playground and continued to experience throughout life, could often be set aside.

### Effervescent experiences

The experience of being with men ‘like’ themselves, who were constructed more as peers than competitors for masculine capital, allowed most participants to enjoy taking part in FFIT, as others have reported in similar circumstances (Robertson *et al*. 2013; Spandler *et al*. 2014). The men joked, teased, supported one another and laughed together about incidents they recounted, as illustrated below:

P4 And the craic was great.

P1 The biggest thing, I think, in life, is laughing because, you know, aw [all] the chemicals go off and you know … […. discussion about a particular night]

P1 But things like that was just, and like, wee bits of banter when we're in the gym [at the club], or playing fitba [football] or that – you'd have a wee craic with somebody. And the laughter, when I played football [in the past]– no’ at a great level, like – but I used to prefer the training bit to the actually going oot [out] and somebody trying to kick lumps out of you. And that was the environment we were working in [on the FFIT programme]. It was almost, coz we had training every week, you know? [Club02_12wkFGD]

P5 It was [like] going to the pub for the banter withoot [without] the drink.

P1 Aye. (Laughing.)

P5 You know? That's what it was. [Club13_12wkFGD]

In these exchanges, participants compare their interpersonal interactions during FFIT to those experienced in other domains: football training earlier in their lives and the pub. Accounts given in focus groups linked this atmosphere to the investment in football culture they shared:

P5 … even before we got into the meat and veg of the group and that, eh, everybody was quite firing back and forward wi’ the banter, straight from the word go, coz football is what we all have, eh? [Club10_12wkFGD]

Data from observations of session deliveries support these recollections. For example, one observation note describes how men responded to encouragement from former club players attending a particular session:

Throughout the time they [former players] speak there are a number of outbreaks of laughter and the men sit attentively and engaged throughout, asking questions toward the end. For some of the men these players were part of the team that they followed as children/teenagers and there is a palpable sense in the room of respect and excitement at their presence. [Club10_ObsWk8]

Similarly, another observation note recorded:

… a great buzz in the room by the time all the men arrive, with lots of laughter and banter, and it was clear that bonds had been formed in previous weeks. [Club10_ObsWk3]

The interactions that took place during FFIT, then, were characterised both by observers and participants as imbued with enjoyment, humour, banter and laughter (‘craic’). These forms of interaction, whether during physical activity or classroom sessions, served to create a ‘buzz’ and ‘excitement’.

These interactions between participants during FFIT sessions can be seen as forms of ‘collective effervescence’. That is, they were moments in which men transcended their individuality through humour, group activities and excitement, through a common passion for football and/or ‘their’ club and a shared desire to lose weight and get fitter. As suggested by Durkheim and Robson, such moments of transcendence can have the effect of constructing, strengthening and renewing the collectively‐grounded identities of those that experience them. The energy that such interactions created, we suggest, was harnessed as men pursued new practices as part of their lifestyle changes.

## Peer support and the (re‐)negotiation of masculine identities

The commonality that participants attributed to one another, strengthened by the ‘effervescence’ experienced through interactions at FFIT sessions, contributed to the formation of groups in which members trusted one another enough to discuss subjects they might feel unable to discuss in other contexts. This process was described in one focus group:

P2 By the same token, there was guys … they sat and owned up, och I had a bad weekend you know. Folk didnae laugh or point fingers, they just said aye, oh fine… you know.

P1 That's just what I was thinking there. I think, again subconsciously because you were with a good group, it didnae matter if you had a bad week, a good week or whatever. You knew that naebody [nobody] was gonnae [going to] take the mick [tease or taunt you] or anything whereas maybe other groups who you didnae feel quite as comfortable wi, mair [more]people are less inclined maybe tae [to] say ‘well, I'm no’ going tae work, I had a shit week or something’ like that. Whereas, when you get on well, I think there's more chance of people… more relaxed so they'll be more open. [Club 11_12wk FGD]

Descriptions of FFIT sessions as spaces of trust and support were also common in observation notes, as illustrated by the following:

Anyone think their diet is not too bad? [question asked by coach] One man says he thinks his is terrible and talks about skipping breakfast and then making bad choices because he's so hungry and eating too much in the evening. Another man refers to this as the ‘caveman diet; everything at once’ and says he does it too. [Club02_ObsWk2]

In the group encounters, then, men were able to reflect on practices they wanted to change collaboratively and without fear of being reprimanded.

Thus, rather than developing into a competition in which men jostled to establish superior achievements to one another, group interactions offered a space in which doxic forms of interaction could be challenged, as illustrated below:

P1 It was funny, listening to men – and I don't want to sound sexist – but men going on about weighing themselves in the morning and what diet they were on and what they were eating… and I think and it was good. And there was a real camaraderie about the course. [Club10_12wkFGD]

An observation note also illustrates the importance of such open discussions during FFIT sessions:

One man says that the motivation that they get from each other is important and the ‘really good discussions’ with ‘like‐minded people’ is helping. There are a lot of nods of agreement from the other men. [Club13_ObsWk7]

In summary, with the support of their peers within the group, FFIT participants constructed group environments in which it became very acceptable to challenge the cultural doxa which usually limit talk of diet and monitoring weight amongst men in other settings, and to openly discuss, accept, support and demonstrate alternative modes of practice in relation to health.

## Performing re‐fashioned masculinities

Our analysis thus suggests that several factors (the creation of culturally bonded groups, through perceived commonalities and shared effervescent experience, and supportive discourses that challenged some potentially‐damaging masculine orthodoxies) allowed men not only to think differently, but also to support each other in making changes to their practices. That is, to enact a changed lifestyle. Observation notes often recorded comments from participants and coaches describing new activities that men had taken up. For example, during a discussion of physical activity over the previous week, one observer recorded that:

[The coach] asks about other activities the men have been doing in the last week apart from walking. Men answer ‐ ‘Cycle’, ‘fives’, a man jokes ‘[I] cannae [can't] count floating in a jacuzzi?!’ [The coach] mentions that there are centres near them that do a variety of activities, for example there is an ice‐rink. One man says that they can do badminton and swimming nearby. [Club4_ObsWk4]

Whilst still the subject sometimes of humorous exchange, regular discussion of the activities that men were doing outside of the programme served to reinforce the new physical activity practices that they were encouraging each other to adopt.

Focus group data also revealed that participants’ discussions of new routines of physical activity were sometimes accompanied by descriptions of new practices of eating and drinking. For example:

P3 I don't drink pints anymore, for a start, and that kind of came out, partly came out when we were talking about calories and making you think aboot it. And I'm no’ daft. I knew there must be mair [more] calories in a pint, but I just couldnae stomach the idea of having a bottle of beer while everybody's getting a pint. And I thought, ‘Right, I'll try this with the bottles of beer’. And then, once I seen the weight coming off, and that, […] So, I went on to the bottles of beer. That changed everything for me, because the pints – you're obviously drinking mair alcohol, for a start, so I'm puggled [tired] come six o'clock when I come hame [home] from the fitba. I only, noo [now], have a few bottles of beer. I've actually started taking the car, coz I've got to the point where I realise I'm no’ even needing a bottle of beer. I'll maybe have one bottle of beer. [Club12_12 week FGD]

Here P3 describes how he was able to supplant his well‐established practice of drinking pints with the new practice of drinking bottled beer to help reduce his calorific intake. Whilst this might seem like a small change, it can be cast as a tactical strategy in response to his friends who were ‘getting a pint’. It also reflects the internalisation of a key message within FFIT, that sustainable changes, even if quite small, can accumulate to effect long term changes in health and health practices.

Another man reflected on the nature of the relationships that enabled such changes to be pursued:

P4 Aye, coz I think the peer, I was going to say pressure, but it's not – it's the reverse of that, but the peer group kind of worked together. Being of a similar size, most of us, and then working down the way – you're seeing, you're almost looking in a mirror. You're seeing the benefit in other people [Club3_12wkFG]

Such examples suggest that the ‘peer’ interactions within FFIT sessions provided some men with a social group in which to rehearse new practices, and with other men. These interactions provided new tactics to add to their performative repertoires as men, including in relation to alcohol. Thus, supported by the interactions that FFIT engendered, men were able to re‐envisage and challenge hegemonic forms of masculinity in ways that supported healthful rather than health‐damaging practices. These examples show how the potentially destructive and often rehearsed link between masculine practices and health‐damaging behaviours, such as excessive drinking, can be decoupled. As we have argued elsewhere (Hunt et al. 2013), the FFIT programme provided men with new scripts for ‘doing health’ whilst still ‘doing gender’ (West and Zimmerman, 1987), or in Butler's (2004) words, ways of ‘undoing’ gender.

## Discussion

Approaches to improving health have often polarised between those that emphasise changing individuals’ behaviours and those that emphasise changing social and economic conditions in which these behaviours are enacted (Baum and Fisher, 2014). The social practice approach we have used here has allowed us to look both ways: towards the social structures of gender relations and to the behavioural routines of consumption and activities. The data we have explored here have demonstrated the powerful backdrop of hegemonic forms of masculinity and the impact they can have on men's vision of themselves and willingness to participate in healthy lifestyle programmes: had Adonis attended FFIT, many men may not have returned for fear of being unable to match up to the hegemon. The data also illustrate the significance of eating, drinking and physical activity behaviours and the awareness these men developed of the role they played in them having gained weight.

However, the approach has enabled us to go beyond the social structure/behaviour dichotomy to consider how both social structures and individual behaviours are mediated through interactions in social networks (Giddens 1987, Pescosolido 1992). The FFIT programme created groups in which both individual behaviours and social orthodoxies could be examined, contested and re‐fashioned. The men could talk about their bodies and the pursuit of weight loss, recognising that this was not normally permitted – as encapsulated in the comment that ‘it was funny listening to men … going on about weighing themselves’. In such ways, the connection between behaviours and the gendered social structure was made, allowing men to engage in the ‘re‐doing’ of both (West and Zimmerman, 2009).

The importance of cultural commonality for the interactions that allowed men to re‐negotiate aspects of their gendered performances and behaviour, must also be emphasised. We have suggested that grounding FFIT in commonalities of habitus (i.e. accumulated experiences of being men with a passion for football, with similar bodies and of similar ages) provided a strong foundation for a supportive group environment in which interactions, and perhaps more ‘inclusive’ performances of masculinity, took place (Anderson 2009). That is to say, the talk and the actions that men generated as groups – such as sharing of old photographs – were made possible by the recognition of having something they enjoyed (the company of other men, football, the club) in common.

The common cultural focus point of the football club, combined with the lively and active group atmosphere, also enabled the production of what we have called, following Durkheim, collective effervescence. As Durkheim argued, and Robson later echoed in relation to football, such collective experiences are energising and confidence‐building. We suggest that the group effervescence that we describe was vital for the successful rehearsal and enactment of new practices; that it provided positive social energy which fuelled confidence and resolve to perform new practices across a range of settings in men's everyday lives.

Other studies that have used professional sports clubs to facilitate and host behaviour change interventions have come to similar conclusions about the important role played by sociality and interaction within these settings. For example, in an article that develops a programme theory for sport‐for‐change programmes for young people, Coalter (2012) suggests that the development of trust, reciprocity and respect between participants (and with coaches) is essential for success. Similarly it has been suggested that ‘positive social interaction was a central mechanism’ that kept men engaged, facilitated change and contributed to increased feelings of wellbeing in initiatives within the Premier League Health (Robertson *et al*. 2013). Spandler *et al*. (2012: 144) noted how men attending a mental health project at a football club saw ‘football clubs as acceptable places to go, in comparison to health care spaces which many saw as stigmatising’; however, they recognise ‘football welfare programmes’ as ‘potential “paradoxical spaces”’ (Spandler *et al*. 2014: 387) in which the use of football culture and clubs as a vehicle for health improvement can be ‘a forum that consciously challenges masculine hegemony’ (Spandler *et al*. 2014: 401) on the one hand, whilst potentially reinforcing gendered practice and perpetuating hegemonic forms of masculinity on the other. In many ways this drove our initial development of the FFIT programme, which consciously drew on the notion of trading of masculine capitals to engage men in a weight loss and healthy living programme. Rather than simply challenging hegemonic forms of masculinity, the FFIT programme allows men to refashion their own masculine identities in relation to specific behaviours and to take part in a collective re‐negotiation of uncritical equations of destructive health practices with gender orthodoxies.

Our study has strengths and limitations. The data were collected in 13 diverse football clubs in Scotland from men from across the socioeconomic spectrum (Hunt *et al*. 2014b). However, the nature of the data we present here, focus group discussions and observation notes as collected, were not amenable to a systematic investigation of differences in accounts by men in different socioeconomic positions. We recognise too that the use of individual interviews might have elicited different accounts in the absence of other participants. Combined with more observations, individual interviews could also have allowed us to explore routine practices, such as eating habits and activity patterns, in more depth and in other social contexts, such as family life and work. This might have allowed for a more sophisticated operationalisation of the social practice approach (Halkier and Jensen 2011). In addition, the use of social network analysis could allow an investigation of the way in which patterns of interaction develop during the course of such programmes; combined with qualitative data on the content of interactions within social networks, this might facilitate a deeper examination of the mechanisms through which wider cultural change to more healthful social practices could be achieved.

Lubans (2014: 1191) suggests that the success of the FFIT programme should encourage ‘researchers and health professionals to use this strategy in other sports (e.g., rugby union, American football, and basketball) to combat the global obesity epidemic’. We claim that the social practice approach we have taken supports the view that such programmes are most likely to work if they bring like‐minded people together (Galdas et al. 2014) and support effervescent group experiences in contexts of symbolic importance; it is partly through the interactions which produce these experiences that lifestyle changes are permitted, and new, more healthful, practices achieved. Thus, we conclude that group interaction that is driven by cultural commonalities can result in ‘effervescent’ experiences which permit and enable the renegotiation of social orthodoxies that often remain tacit and unspoken, and which shape behaviour in daily life. Interventions that facilitate such interactions and experiences should be designed and assessed for potential public health collaborations in other social and cultural domains. As well as other sports settings, these domains could include faith‐based organisations, work‐places in which people have strong commitment to the ‘firm’ or employing organisation, and clubs which inculcate strong bonds between members, such as Rotary Clubs, youth clubs, and hobby groups.

## Conclusion

We argue that taking a social practice approach, which recognises the structural, individual and interaction‐based aspects of life, has enabled us to investigate the appeal and success of FFIT in such a way as to illuminate the multiple social processes through which the programme is able to support participants’ lifestyle change and weight loss. We demonstrate the importance of shared habitus, effervescent experiences and social support which allowed and supported the adoption and performance of new (masculine) health practices. As social scientists strive to contribute more to the development of public health interventions, the critical role of interaction in the formation and change of health practices needs to be prioritised.

## Competing interests

The authors declare that they have no competing interests.

## Authors’ contributions

CB conceived the paper and led the qualitative analysis with support and input from KH, CMG and SW. AMc and Susan Smillie led data collection at the club stadia, with support from KH, CMG, and SW. KH, CMG, CB and SW read transcripts. CB drafted the manuscript. All authors commented on drafts, and read and approved the final manuscript.
